# Data Science for Extubation Prediction and Value of Information in Surgical Intensive Care Unit

**DOI:** 10.3390/jcm8101709

**Published:** 2019-10-17

**Authors:** Tsung-Lun Tsai, Min-Hsin Huang, Chia-Yen Lee, Wu-Wei Lai

**Affiliations:** 1 Institute of Manufacturing Information and Systems, National Cheng Kung University, Tainan 701, Taiwan; jkbon49027@gmail.com; 2 Department of Surgery, National Cheng Kung University Hospital, Tainan 701, Taiwan; icu.tainan@gmail.com (M.-H.H.); lai.wuwei@gmail.com (W.-W.L.); 3 College of Medicine, National Cheng Kung University, Tainan 701, Taiwan

**Keywords:** extubation, surgical intensive care unit, data mining, machine learning, precision medicine

## Abstract

Besides the traditional indices such as biochemistry, arterial blood gas, rapid shallow breathing index (RSBI), acute physiology and chronic health evaluation (APACHE) II score, this study suggests a data science framework for extubation prediction in the surgical intensive care unit (SICU) and investigates the value of the information our prediction model provides. A data science framework including variable selection (e.g., multivariate adaptive regression splines, stepwise logistic regression and random forest), prediction models (e.g., support vector machine, boosting logistic regression and backpropagation neural network (BPN)) and decision analysis (e.g., Bayesian method) is proposed to identify the important variables and support the extubation decision. An empirical study of a leading hospital in Taiwan in 2015–2016 is conducted to validate the proposed framework. The results show that APACHE II and white blood cells (WBC) are the two most critical variables, and then the priority sequence is eye opening, heart rate, glucose, sodium and hematocrit. BPN with selected variables shows better prediction performance (sensitivity: 0.830; specificity: 0.890; accuracy 0.860) than that with APACHE II or RSBI. The value of information is further investigated and shows that the expected value of experimentation (EVE), 0.652 days (patient staying in the ICU), is saved when comparing with current clinical experience. Furthermore, the maximal value of information occurs in a failure rate around 7.1% and it reveals the “best applicable condition” of the proposed prediction model. The results validate the decision quality and useful information provided by our predicted model.

## 1. Introduction

Extubation decision is critical during a surgical intensive care unit (SICU) stay. Assessment of a patient’s readiness for removal of the endotracheal tube in the intensive care unit (ICU) is usually based on respiratory, airway, neurological measures, etc. Extubation is mostly decided right after a weaning readiness test involving spontaneous breathing trial (SBT) or low levels of assisted ventilation. Even among patients who meet all weaning criteria and successfully perform a weaning readiness test, 10 to 20% still experience extubation failure (EF) [1]. Patients who suffer EF are usually associated with extremely poor outcomes, including high probability of mortality from 25 to 50% [2].

The extubation failure is defined as inability to sustain spontaneous breathing after removal of the artificial device such as an endothracheal or tracheostomy tube, and need for reintubation within a prespecified time window ranging from 24 h to one week [3]. The reasons for EF are diverse and often short of recognition. There is usually a significant respiratory distress episode accompanied with reintubation, which may be related to primary respiratory failure, congestive heart failure, airway secretion accumulation or upper airway obstruction. This multiplicity of causative factors contributes to explain the clinical difficulties raised by extubation and the persistent uncertainties about the pathophysiology of EF. Given the many causes for EF, data centered only on respiratory physiology may fail to constitute a reliable guide for decision making in extubation.

Endotracheal extubation refers to the removal of an endotracheal tube from the trachea. This procedure is commonly performed in operating rooms, post anesthesia care units or ICU. However, the decision to extubate must be made carefully, particularly because respiratory and airway-related complications are more likely to occur after extubation than after intubation and even cause death. Clinically, endotracheal extubation is usually contraindicated when the patient’s ability to protect the airway is impaired or when the patient cannot maintain adequate spontaneous respiration. Miu et al. [4] listed the criteria for endotracheal extubation such as heart rate, blood pressure, Glasgow coma scale, FiO_2_, SpO_2_, PaO_2_, PaCO_2_, pH, rapid shallow breathing index (RSBI), tidal volume (VT), respiratory rate (RR), etc.

There are several causes of EF. While few extubation-related complications are life threatening, hypoxemia is the common one related to severe complications [5]. In the period after extubation, early respiratory insufficiency may be caused by poor ventilation or residual neuromuscular blockade. Acute upper-airway obstruction may be caused by laryngospasm, especially in children. Vocal-cord dysfunction is a rare cause of airway obstruction and sometimes requires immediate reintubation. Incidence of EF usually varies between 6% and 47% [6]. Epstein et al. found that patients who need reintubation had significantly increased duration of mechanical ventilation and prolonged ICU stay [7]. Assessment of patients’ readiness for extubation in the ICU is based on several measurements such as respiratory, blood, biochemistries and neurological measures. However, nearly 20% of these patients still need reintubation. To resolve the issue, there are several approaches to evaluate the status of each patient and ICU also provides a training procedure before extubation. However, it is difficult to identify the best way for all situations.

While different clinical approaches have been conducted on EF, not all patients recognize the risk of reintubation equally and the physiopathology of EF is not fully clarified. Thus, our knowledge of the best ways to prevent and manage EF is still limited. In the past few decades, many studies have been conducted to assess the state of the patient before performing extubation. In addition to traditional statistical analysis, some techniques of machine learning have arisen recently. In literature, there are four types of methods generally applied to the decision making on extubation. They are statistical analysis [8], acute physiologic and chronic health evaluation (APACHE) score and sequential organ failure assessment (SOFA) [2,9,10,11,12], machine learning [13,14,15] and Bayesian decision [16,17,18]. However, the approaches mentioned above are applied independently and non-comprehensively. Typically, the hospitals rely on the RSBI and their clinical experience to make an extubation decision on a case-by-case basis. This decision is not supported by a systematic analysis.

This study aims to propose a data science framework to identify the important variable, enhance the accuracy on the prediction of extubation and investigate the value of information provided by our prediction model. The proposed framework addressed the data imbalance problem, which provide an unexpected excellent prediction accuracy when the number of samples in the majority class and the minority class present an extreme imbalance. Therefore, our proposed framework is systematic and comprehensive to enhance the quality of the extubation decision.

## 2. Data Science Framework

**Figure 1:**
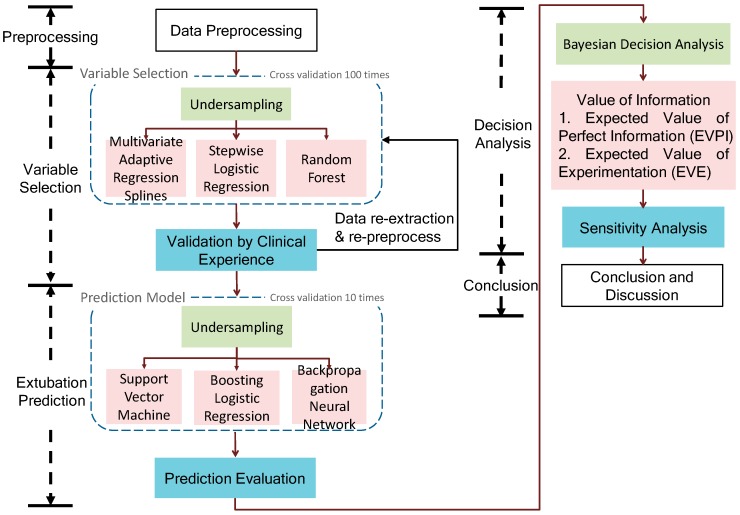
The data science framework of endotracheal extubation.

This section describes the proposed data science framework and its methodologies. Figure 1 shows that a proposed data science framework of endotracheal extubation involves data preprocessing, variable selection, extubation prediction and Bayesian decision analysis. Finally, the framework identifies the significant variables, uses them to build the prediction model and investigates the value of information regarding an extubation decision.

### 2.1. Data Preprocessing

Data preprocessing is used to deal with the incomplete or inconsistent dataset collected from diverse information systems in the ICU. Data preprocessing enhances the data quality which significantly affects the performance of the prediction model. The data is collected from IntelliVue Clinical Information Portfolio (ICIP) including patient data and several electronic health records. We first remove the null and redundant columns and then combine the patients’ ID with their corresponding time they entered the ICU as a unique key (i.e., ID) for binding all the data sheets. Second, we address each data sheet by variable combination and data type transformation. Then, we merge each data frame according to the key ID. Based on the previous studies [1,19] and the experts’ instruction, for one specific observation we only select the related data within the 48 h before or after extubation. Next, we transformed the categorical data into binary (i.e., dummy) variables to fit some machine learning models. Finally, we use the variance inflation factor (VIF) to address the collinearity problem potentially resulting in wrong identification of relevant predictors in statistical models [20,21,22]. We do stepwise procedure to remove one highly-correlated variable every iteration, and these removed variables are sent to clinical validation.

### 2.2. Variable Selection and Prediction Model

The variable selection and extubation prediction are described. First, due to the data imbalance problem (i.e., number of successes is much larger than number of failures), the under-sampling technique is suggested. In particular, we keep all failure cases and randomly sample the same number of failure cases from success cases for making the success–failure ratio equal to 1. Thus, we generate a balance dataset for variable selection and extubation prediction and we repeat the data generating process (DGP) 100 times for repeated random sub-sampling validation (i.e., Monte Carlo cross-validation).

Variable selection is a method to select important variables (or remove the redundant or insignificant factors) in order to (1) avoid the curse of dimensionality which may lead to computational complexity and poor performance of the prediction model; (2) provide a better understanding of the causal relationship between predictors and response variable; (3) suggest a cost-effective monitoring with fewer control charts regarding these important variables [22]. In this phase, we suggest three variable selection techniques (including linear and nonlinear models)—Multivariate adaptive regression splines (MARS), stepwise logistic regression (SLR), random forest (RF)—to rank the relevant importance of factors. The reason we suggest three techniques is because we are not familiar with the geometric relation and property between predictors and response variable in the dataset. Thus we apply three methods rather than one and suggest (1) total frequency (TF) of the selected variables by three techniques [23]; (2) 100 times sampling cross-validation for the robustness to identify the important variables. In addition, because we merged each data frame according to the corresponding ID, the number of observations decreased dramatically since some patients’ IDs do not match others from a variety of data sheets. Thus, we suggest repeating the data merger again with respect to the selected variables to increase the number of samples. In our case study, the number of observations approximately doubled after data re-merger.

Finally, we use these selected variables to construct the extubation prediction models, including support vector machine (SVM), boosting logistic regression (BLR) and backpropagation neural network (BPN), and assess the performance of each prediction model by the confusion matrix for each method. Due to a relatively small testing dataset, 10 times sampling cross-validation is used. For performance benchmarking, we also compare the proposed framework to the single index RSBI and APACHE II commonly used in clinical practice.

### 2.3. Bayesian Decision Analysis

Based on the extubation prediction model mentioned above, this section uses the prediction results to enhance the extubation decision by applying Bayesian decision analysis and assessing the value of information provided by the prediction model. Bayesian analysis is a method to modify the probability (posterior) by collecting the observed results from the uncertain event. Since the Bayesian analysis estimates the posterior probability (i.e., given an observed event, it estimates the probability of the hypotheses/population/unknown parameters that may explain the observed data), we can treat the probability distribution of collected dataset as a prior distribution (i.e., it gives the probability of observed data for a given hypothesis) and the extubation prediction result in the testing dataset as the likelihood function (i.e., it quantifies the possibility that the observed data would have been observed as a function of the hypotheses). We summarize the Bayesian inference as investigating an uncertain event as an unverified hypothesis presented by $\tilde{\theta }$ and all the possible results (i.e., state of nature space) are $\theta_{1},\;\theta_{2},\ldots ,\theta_{m}$. Let set $P\left(\tilde{\theta }=\theta_{j}\right)$ be the prior probability of $\tilde{\theta }=\theta_{j}$, that is, the probability given by the dataset without further information. When the sample space $x_{i}$ is observed by the decision maker, the probabilities under the given $x_{i}$ are corrected for each $\theta_{j}$ based on the Bayesian theorem’s so-called posterior probability. That is,

$$P\left(\tilde{\theta }=\theta_{j}|x_{i}\right)=\frac{P\left(\theta_{j}\cap x_{i}\right)}{P\left(x_{i}\right)}=\frac{P(x_{i}|\tilde{\theta }=\theta_{j})\times P\left(\theta_{j}\right)}{\sum_{j=1}^{m}P(x_{i}|\tilde{\theta }=\theta_{j})\times P\left(\theta_{j}\right)}$$

where $P(x_{i}|\tilde{\theta }=\theta_{j})$ is the likelihood function of $\tilde{\theta }=\theta_{j}$ when $x_{i}$ occurred (i.e., when we observe the sample $x_{i}$, the probability that $\tilde{\theta }$ is equal to the $\theta_{j}$). Here, we introduce a new idea and replace all the observed data and likelihood function by the predicted results from the prediction model. That is, this study assumes that we believe the prediction model. Thus, based on the Bayesian decision analysis, we can enhance the decision quality and integrate the data science technique into the decision framework (i.e., the proposed framework as Figure 1).

From the previous phase, the BPN is suggested for estimating the likelihood function due to a higher accuracy of prediction. The decision tree and Bayesian analysis are used to enhance the decision quality and quantify the value of information presented by the expected monetary value (EMV) and the expected value of experimentation (EVE) [24]. The decision tree presents a tree structure which can display the details concerning the status in the decision process and demonstrates all possible actions that the decision maker would take and use the probability to show the possible scenarios for the uncertain factors. We calculate the expected profit/loss value of all possible actions for supporting decision-making. The performance of each node is usually characterized by expected monetary value (EMV), which can be calculated by the folding-back method and we select the best one and its corresponding decision node as the value for the next backward iteration. Finally, we conduct a sensitivity analysis of failure rate to assess the value of information provided by the prediction model and identify the best failure rate that can maximize the value of information to validate our proposed framework. Note that this study assesses the value of information from the “cost” aspect and thus we aim to minimize the expected loss.

## 3. Data and Results

Because all data being used in the study were part of routine clinical practice, the protocol was approved by the Institutional Review Board of National Cheng Kung University Hospital (approval no.: B-ER-105-362) with a waiver of informed consent. In our empirical study, the data is collected from IntelliVue Clinical Information Portfolio (ICIP) including patient data and several electronic health records from October 2015 to September 2016. The imbalanced panel datasets including several tables regarding biochemistry, arterial blood gas (ABG), blood cell, Glasgow coma scale (GCS), APACHE, extubation, etc. are collected from different information systems, in our case hospitals. There are 23 variables with the number of observations being between 1565 and 626,894. Through data preprocessing, the processed data (i.e., several data sheets combined into a single table) is with 359 observations including 49 failure cases (i.e., reintubation).

Table 1 shows the patients’ characteristics. It shows that APACHE II indeed presents a significant difference between success and failure of extubation. The cross validation with 100 times the result of variable selection is shown in Table 2 and we find both MARS and SLR have similar results; however, RF shows difference in some variables such as eye opening, RSBI and $pO_{2}\_FiO_{2}$.

Based on a scree plot we select eight important variables suggested by TF, we then repeat the data preprocessing (i.e., re-preprocess) to expand the number of observations to 704 based on these eight variables and then build SVM, BLR and BPN for extubation prediction. The hyperparameters of SVM, BLR and BPN are optimized by the grid search and cross validation. Note that based on 20% data for testing and success–failure ratio equal to 1 for data balance, we randomly choose 10 from 49 failure cases and 10 from 655 success cases for building testing datasets. The prediction results of different models in the testing dataset were shown in Table 3 via 10-time cross validation. We also list BLR and BPN with single index RSBI and APACHE II to compare with typical methods used in practice (SVM with single index is ignored due to its relatively poor performance with selected variables by TF).

In fact, the penalty in false positive (i.e., predict success in extubation but actually fail) is more serious than false negative (i.e., predict failure in extubation but actually succeed). In fact, there is a trade-off between false positive and false negative, thus we aim to select the prediction model with high accuracy and low false positive. In Table 3, BPN with selected variables by TF shows better performance. Note that this study did not suggest BPN with APACHE II as a prediction model since zero false positive is too ideal.

**Figure 2:**
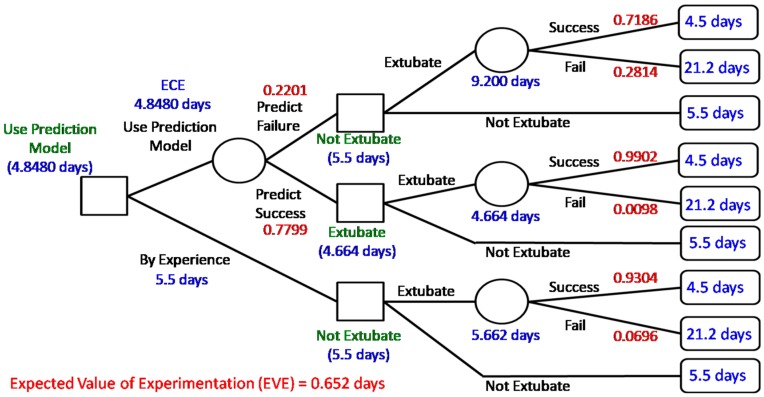
Results of Bayesian decision analysis and value of information.

According to the prediction result of BPN, the value of information provided by the data science prediction model can be investigated by Bayesian decision analysis. In particular, building a decision tree shows the status along with the expected cost/loss after the decision of extubation as shown in Figure 2. Comparing with successfully extubated patients, the patients who need reintubation are more likely to spend more time in the ICU and the intubated patients should spend one more day to recheck the status before extubation [5]. Thus, in our case hospital, the cost (also called penalty/loss) is characterized by “more time-spent in ICU (i.e., more days of stay in the ICU)” shown in the right-hand side of Figure 2.

Figure 2 shows the calculated expected cost (i.e., days of stay in the ICU) of each chance node for decisions made. When the case of perfect information is considered, the expected cost is equal to 4.5696 days (expected costs under perfect information, ECPI); however, the expected cost without extra information (ECWI) is 5.662 days. Based on clinical experience, the cost of deciding not to extubate was 5.5 days less than 5.662 and we make the decision to “not extubate”. Thus, the expected value of perfect information (EVPI) is EVPI=5.5−4.5696(ECPI)= 0.9304 (days). That is, if we have perfect information, we will on average save 0.9304 days (patients staying in the ICU) when making an extubation decision. In addition, the expected cost of using the prediction model (i.e., expected costs of experiment, ECE) on an extubation decision is 4.8480 days less than 5.5 days and thus we suggest the decision use prediction model”. Therefore, the expected value of experimentation (EVE) is calculated as EVE=5.5−4.8480= 0.652 (days). That is, using the prediction model will roughly save 0.652 days (patient staying in the ICU) compared with current clinical experience. It implies useful information provided by the prediction model and validates the proposed data science framework. Note that we ignore the cost of building a prediction model since it is relatively small.

**Figure 3:**
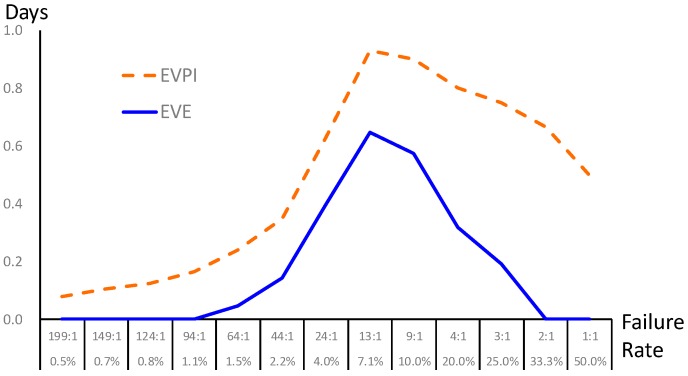
Sensitivity analysis of the failure rate regarding prior probability.

Finally, a sensitivity analysis is conducted to characterize the uncertain events in the decision-making process in Figure 2. The failure rate is regarded as the prior probability provided by the prediction model (i.e., BPN) in this study. Since the failure rate in extubation usually ranges from 2% to 47% in the literature (the failure rate collected from our case hospital is 0.0696 after re-preprocess, i.e., success-versus-failure is about 13:1), we performed sensitivity analysis of the failure rate from 0.5% to 50%; that is, we consider different scenarios in general hospitals and validate the value of information. The failure rate directly affects the prior shown as the bottom branch in Figure 2. The EVPI and EVE are calculated as Figure 3. The result shows that our proposed framework is superior with positive EVE when the failure rate is between 1.5% and 25%; in particular, the maximal EVE occurs in a failure rate around 7.1%. At the moment, the proposed data science framework shows the best value of information just like our case study. On the other hand, though BPN provides prediction with high accuracy, the proposed framework may not be helpful when the failure rate is lower than 1.1% or over 33.3%.

## 4. Discussion

More and more machine learning techniques are used in medical care, in particular, ICU [25]. This study focuses on extubation prediction. In literature, weaning parameters such as tidal volume, minute ventilation, maximum expiratory pressure, etc. are used to support the weaning process; however, they may not support predicting extubation well [26]. In addition, there are several criteria such as APACHE, RSBI and SOFA to support the extubation decision; however, the contribution is limited since the single index, constructed with several variables or partially distinct variables, did not provide a comprehensive view for extubation decisions. This study proposes a data science framework including variable selection, a prediction model and Bayesian decision analysis to support the extubation decision. The framework identifies the significant variables related to the endotracheal extubation by MARS, SLR and RF, and then provides excellent prediction performances by SVM, BLR and BPN. The results are compared with the current indices such as APACHE II and RSBI. In particular, the variable selection phase suggest that APACHE II and WBC are two critical factors affecting EF. Prediction with the BPN model provides high accuracy, and this result is consistent with previous studies, which reported that the predictive performance of artificial neural networks (ANNs) was better than those of RSBI and maximum expiratory pressure [26] and better than those of RSBI and maximal inspiration pressure (PIMAX) [27]. In previous studies, the factors that affect the EF are APACHE II, RSBI, sex, creatinine, PIMAX, ABG, etc. [3,4,26,27,28]. All these factors are included in our data science framework and thus it provides a robust prediction based on comprehensive information. Finally, the predictive results of BPN are used for Bayesian decision analysis. This phase is critical to provide a connection from predictive analytics to prescriptive analytics [29]; that is, data science not only provides a model for prediction but also enhances the decision-making process in practice by investigating the value of information and decision risk (i.e., days of stay in the ICU) [30]. The results, showing a positive value of information, enhance confidence in applying data science for supporting extubation decisions in clinical practice (i.e., the prediction model will roughly save 0.652 days of a patient staying in the ICU). In fact, the space and beds in SICUs are limited and to shorten the patient’s stay in the SICU will improve the bed turnover rate and the service quality. The most interesting thing derived from this study is that the maximal value of information occurs in a failure rate around 7.1%. This reveals the “best applicable condition” of the proposed prediction model.

## 5. Conclusions

This study proposes a data science framework for extubation prediction and quantifies the value of information. The analysis results validate the decision quality and useful information provided by our predicted model. The proposed data science framework is general and can be extended to other cohorts of ICU patients (e.g., medical ICU, neuro ICU, cardiac ICU, etc.) by collecting specific and related factors. In fact, the methodologies of variable selection and prediction models are more generalized to other industries (e.g., manufacturing and services) while decision analysis needs to specify the limitation and practical experience based on the applied domain. For further research, besides the factors regarding biochemistry, arterial blood gas (ABG), Glasgow coma scale, etc., there are several environmental factors (e.g., family care, time interval of doctor’s review, etc.) and neurological dysfunctions (e.g., dysphagia) [31] which can be considered to improve the extubation decision and patient’s status.

## Figures and Tables

**Table 1:** Patients’ characteristics with some features.

	Success (Mean/Std)	Failure (Mean/Std)
APACHEII	12.11/5.21	17.82/5.80
RSBI	48.04/27.91 breath/(min × L)	67.88/34.41 breath/(min × L)
Heart Rate	91.49/17.06 bpm	94.69/14.91 bpm
White Blood Cells	12.05/4.85 10^3^/μL	12.87/4.37 10^3^/μL
Na^+^	138.47/4.29 mmol/L	140.98/6.69 mmol/L
Glu	175.47/62.66 mg/dL	182.14/55.54 mg/dL
PaO_2_/FiO_2_	367.78/96.62 mmHg	324.26/75.72 mmHg
Hct (ABG)	34.16/5.35%	31.64/4.04%
Age	58.83/15.40	64.58/16.89
Weight	64.15/14.77 kg	62.03/13.12 kg

**Table 2:** The results of variable selection methods.

Multivariate Adaptive Regression Splines		Stepwise Logistic Regression		Random Forest		Total Frequency	
Variables	Freq.	Variables	Freq.	Variables	Freq.	Variables	Freq.
ApacheII	98	ApacheII	94	ApacheII	100	ApacheII	292
Eye_Opening	42	Eye_Opening	64	WBC	74	WBC	155
WBC	41	WBC	40	Glu	59	Eye_Opening	114
Heart_Rate	36	RSBI	32	Na	58	Heart_Rate	111
Glu	30	Hct (ABG)	25	Heart_Rate	54	Glu	108
Na	30	Heart_Rate	21	Hct (ABG)	53	Na	103
RSBI	25	Glu	19	$pO_{2}\_FiO_{2}$	38	Hct (ABG)	100
Platelets	24	Na	15	Weight	36	RSBI	90
Gender_men	24	PT_INR	11	ARTmean_BP	35	Platelets	64
Hct (ABG)	22	Verbal_Response	9	PT_INR	35	Weight	62
Verbal_Response	19	Gender_men	9	Platelets	33	Verbal_Response	61
Weight	17	Weight	9	RSBI	33	PT_INR	59
ARTmean_BP	13	Platelets	7	Verbal_Response	33	ARTmean_BP	54
PT_INR	13	ICU_Emergency	7	PIMAX	32	$pO_{2}\_FiO_{2}$	53
$pO_{2}\_FiO_{2}$	12	ARTmean_BP	6	Eye_Opening	8	PIMAX	44
ICU_Emergency	12	PIMAX	5	Gender_men	3	Gender_men	36
PIMAX	7	$pO_{2}\_FiO_{2}$	3			ICU_Emergency	19

**Table 3:** A comparison of extubation prediction by different models.

Performance Metrics	SVM (TF)	BLR (TF)	BPN (TF)	BLR (RSBI)	BLR (APACHE II)	BPN (RSBI)	BPN (APACHE II)
True Positive	6.2	8.1	8.3	5.1	7.8	8	7.2
False Negative (Type II error)	3.8	1.9	1.7	4.9	2.2	2	2.8
False Positive (Type I error)	1.1	1.8	1.1	5	2.6	2.7	0.0
True Negative	8.9	8.2	8.9	5	7.4	7.3	10.0
Sensitivity	0.620	0.810	0.830	0.510	0.780	0.80	0.72
Specificity	0.890	0.820	0.890	0.500	0.740	0.73	1.00
Accuracy (Std. Dev.)	0.755 (0.019)	0.815 (0.018)	0.860 (0.016)	0.505 (0.029)	0.760 (0.030)	0.765 (0.017)	0.860 (0.012)

## Abbreviation

ABG	arterial blood gas
ANN	artificial neural network
APACHE II	acute physiology and chronic health evaluation (APACHE) II score
BLR	boosting logistic regression
BPN	backpropagation neural network
DGP	data generating process
DT	decision tree
ECE	expected costs of experiment
ECPI	expected costs under perfect information
ECWI	expected cost without extra information
EF	extubation failure
EMV	expected monetary value
EVE	expected value of experimentation
EVPI	expected value of perfect information
GCS	Glasgow Coma Scale
ICIP	IntelliVue Clinical Information Portfolio
ICU	intensive care unit
MARS	multivariate adaptive regression splines
PIMAX	maximal inspiration pressure
PT_INR	prothrombin time_international normalized ratio
RF	random forest
RR	respiratory rate
RSBI	rapid shallow breathing index
SBT	spontaneous breathing trial
SLR	stepwise logistic regression
SOFA	sequential organ failure assessment (SOFA) score
SVM	support vector machine
VIF	variance inflation factor
VT	tidal volume
WBC	white blood cells
